# Management of multiple brain metastases: a patterns of care survey within the German Society for Radiation Oncology

**DOI:** 10.1007/s11060-021-03714-w

**Published:** 2021-02-23

**Authors:** Johannes Kraft, Michael Mayinger, Jonas Willmann, Michelle Brown, Stephanie Tanadini-Lang, Lotte Wilke, Matthias Guckenberger, Nicolaus Andratschke

**Affiliations:** University Hospital Zurich: UniversitatsSpital Zurich, Zurich, Switzerland

**Keywords:** Brain metastases, Multiple brain metastases, Stereotactic radiotherapy, Radiosurgery, Pattern of care, Management of brain metastases

## Abstract

**Purpose:**

The treatment of brain metastases (BM) has changed considerably in recent years and in particular, the management of multiple BM is currently undergoing a paradigm shift and treatment may differ from current guidelines. This survey was designed to analyze the patterns of care in the management of multiple BM.

**Methods:**

An online survey consisting of 36 questions was distributed to the members of the German Society for Radiation Oncology (DEGRO).

**Results:**

In total, 193 physicians out of 111 institutions within the German Society for Radiation oncology responded to the survey. Prognostic scores for decision making were not used regularly. Whole brain radiotherapy approaches (WBRT) are the preferred treatment option for patients with multiple BM, although stereotactic radiotherapy treatments are chosen by one third depending on prognostic scores and overall number of BM. Routine hippocampal avoidance (HA) in WBRT is only used by a minority. In multiple BM of driver-mutated non-small cell lung cancer origin up to 30% favor sole TKI therapy as upfront treatment and would defer upfront radiotherapy.

**Conclusion:**

In multiple BM WBRT without hippocampal avoidance is still the preferred treatment modality of choice regardless of GPA and mutational status, while SRT is only used in patients with good prognosis. Evidence for both, SRS and hippocampal avoidance radiotherapy, is growing albeit the debate over the appropriate treatment in multiple BM is yet not fully clarified. Further prospective assessment of BM management—ideally as randomized trials—is required to align evolving concepts with the proper evidence and to update current guidelines.

## Introduction

Metastatic spread to the CNS is a common scenario in many metastasized solid cancers like breast, lung, renal carcinoma or melanoma, with a cumulative risk of 10–30% in adults [1, 2]. Untreated, brain metastases (BM) remain a substantial source of morbidity and mortality and leads to neurocognitive and functional deficits [3, 4]. Historically, BM have been treated with whole-brain radiotherapy (WBRT) [5]. A treatment of the whole brain and possible microscopic spread was considered necessary to prevent new metastases. WBRT continues to be used today, but is considered obsolete in patients with limited brain metastases where national and international guidelines provide a clear recommendation to treat patients either with stereotactic radiotherapy (SRS) or surgery based on results of phase III randomized trials [6–9]. In contrast to a limited number of BM, the current guidelines provide no clear support for decision making in multiple BM (> 4) and give a broad scope of choice. However, this issue is becoming increasingly important as, in addition to a general increase in the incidence of brain metastases, in particular that of multiple lesions is increasing [10, 11].

Due to technical progress, stereotactic radiotherapy has also evolved as a possible treatment option in multiple brain metastases as well as whole brain irradiation with hippocampal sparing to minimize deterioration in neurocognition with sparing of the neuronal stem cell compartment. Furthermore, a tremendous progress in the development of new systemic agents especially in driver-mutated NSCLC have led to promising targeted systemic therapy options. Newer generations of tyrosine-kinase-inhibitors seem to be well effective in the brain and consequently raise the question of whether to use upfront radiation therapy or upfront TKI or both [12].

Overall, generated clinical data is currently still limited and high level evidence is still evolving; however, due to the improvement in prognosis through targeted therapies, the management of multiple BM has become increasingly important. To address this highly relevant topic, a survey was conducted to clarify the role of different treatment options and considerations. Additionally, we tried to analyze general management in stereotactic treatment of BM, patient selection and to capture a snapshot of therapy concepts currently being used in German-speaking countries.

## Methods

After a literature search and based on our institutional treatment policy, a survey was developed from a collection of questions from physicians at the University Hospital in Zurich. In the selection of topics, we tried to focus on relevant issues in the treatment of multiple BM. The survey with either single choice or multiple choice questions was implemented with the online platform “SurveyMonkey” and was designed to be completed in around 15 to 20 min. Questions to the following topics were queried: general information on staff and institution; technical implementation of stereotactic radiotherapy, treatment application, follow-up and specific scenarios with image samples in patients with multiple BM from non-small cell lung cancer (NSCLC). The survey was confidential and completely anonymous. This allowed the participants to express their personal and professional opinion, even if it did not comply with the national and international guidelines.

After internal validation by an independent radiation oncologist the questionnaire was distributed to the members of the German Society for Radiation Oncology (DEGRO) via email with a link to the online survey in December 2018. After 8 weeks a reminder email was sent.

For the final evaluation, responses of residents were excluded and only responses from specialists were considered in order to reduce the heterogeneity of the respondents. Frequency distributions of responses for each question were calculated. The data was analyzed by type of institution (academic (university hospital) vs. non-academic institution (general hospital, medical care center or practice), high-volume center (defined as > 50 patients with BM per year) vs. low-volume center and also by individual position within the department (head/deputy head of department vs. senior physician/consultant) using Pearson’s chi-squared test. A p-value of < 0.05 was considered as significant.

## Results

### Institutional and personal characteristics

In total, answers were received from 193 participants from 111 institutions, which corresponds to at least 24% of the total number of Centers within the German Society for Radiation Oncology [13]. In the end, 171 responses were available for final evaluation by excluding responses from residents (n = 22). About half of the answers came from physicians working in hospitals (22% university hospitals and 28% non-academic hospitals). At least 70% of the participants worked in centers with more than 5 physicians. Regarding the function within the department, our survey was answered by 34 heads of department/Chief Physicians and again 34 physicians working in leading positions within the department (Deputy Head/Chairman, Deputy Chief Physician) representing almost 40% of all respondents. The results of survey respondent demographics are presented in Table 1.

**Table 1:** Demographics of respondents and their affiliated centers

	n	%
Overall	*171*	*100*
Institutional characterization		
Academic Institution (University-Hospital)	37	21.6
Non-academic hospital	47	27.5
Medical Care Center	42	24.6
Practice	45	26.3
Position at work		
Head of department	34	19.9
Deputy head of department	34	19.9
Senior Physician	52	30.4
Consultant	51	29.8
Physicians per center		
1–5 physicians	51	29.8
6–10 physicians	55	32.2
11–15 physicians	27	15.8
≥ 16 physicians	38	22.2
Treated patients per year (per center)		
< 10 patients	8	4.7
10 to 50 patients	86	50.3
50 to 100 patients	49	28.7
> 100 patients	28	16.4

More than 90% stated that the therapy decision was based on an individual tumor board decision in every case or at least in most cases (> 50% of all cases). Decisions in university hospitals or high-volume centers were statistically more often based on recommendations from interdisciplinary tumor boards when compared to non-academic centers or low-volume-centers (p-value ≤ 0.001 and p-value = 0.022, see Table 2).

**Table 2:** Statistical analysis of differences in treatment policies stratified by type of institution or position

Question and answers	All answers	Academic centers (University Hospitals) (A)	Non-academic centers (B)	Head/deputy head of department (A)	Others (B)	High-volume centers < 50 pts/year (A)	Low-volume centers > 50 pts/year (B)
Max total no. = 171	n (%)	n (%)	n (%)	n (%)	n (%)	n (%)	n (%)
		p-value*		p-value*		p-value*	
Do you use a prognostic score before treatment of brain metastases?							
Yes	64 (37.4)	20 (54.1)	44 (32.8)	15 (44.1)	49 (35.8)	34 (44.2)	30 (31.9)
No	107 (62.6)	17 (45.9)	90 (67.2)	19 (55.9)	88 (64.2)	43 (55.8)	64 (68.1)
		***0.018***		0.368		0.100	
Does the prognostic score influence your decision regarding therapeutic options?							
Yes	62 (36.8)	20 (54.1)	43 (32.1)	14 (41.2)	49 (35.8)	32 (41.6)	31 (33.0)
No	108 (63.2)	17 (45.9)	91 (67.9)	20 (58.8)	88 (64.2)	45 (58.4)	63 (667.0)
		***0.014***		0.558		0.247	
Is the treatment recommendation for cerebral irradiation made in an interdisciplinary tumor board?							
Yes, always	53 (31.0)	21 (56.8)	32 (23.9)	12 (35.3)	41 (29.9)	32 (41.6)	21 (22.3)
Yes, in most cases (> 50%)	105 (61.4)	16 (43.2)	89 (66.4)	19 (55.9)	86 (62.8)	41 (53.2)	64 (68.1)
No, not regularly	13 (7.6)	0 (0)	13 (9.7)	3 (8.8)	10 (7.3)	4 (5.2)	9 (9.6)
		** < 0.001**		0.761		***0.022***	
Regarding RPA-Score: At which score would you perform a stereotactic irradiation?							
No use of RPA	106 (62.0)	17 (45.9)	89 (66.4)	19 (55.9)	87 (63.5)	44 (57.1)	62 (66.0)
Only RPA 1	12 (7.0)	5 (13.5)	7 (5.2)	4 (11.8)	8 (5.8)	5 (6.5)	7 (7.4)
RPA 1 and 2	41 (24.0)	13 (35.1)	28 (20.9)	6 (17.6)	35 (25.5)	26 (33.8)	15 (16.0)
RPA 1–3	12 (7.0)	2 (5.4)	10 (7.5)	5 (14.7)	7 (5.1)	2 (2.6)	10 (10.6)
		0.059		0.116		***0.018***	
Regarding GPA-Score: At which score would you perform a stereotactic irradiation?							
No use of GPA	109 (63.7)	17 (45.9(	92 (68.7)	20 (58.8)	89 (65.0)	46 (59.7)	63 (67.0)
GPA > 2.5	39 (22.8)	14 (37.8)	25 (18.7)	8 (23.5)	31 (22.6)	22 (28.6)	17 (18.1)
GPA < 2.5 and > 2.5	23 (13.5)	6 (16.2)	17 (12.7)	6 (17.6)	17 (12.4)	9 (11.7)	14 (14.9)
		***0.026***		0.695		0.257	
What is the main treatment choice in ≥ 75% of cases in patients presenting with 4–10 brain metastases and a GPA < 2.5?							
SRS or fSRT	32 (18.7)	16 (43.2)	16 (11.9)	9 (26.5)	23 (16.8)	22 (28.6)	10 (1.6)
WBRT ± boost or HCS	139 (81.3)	21 (56.8)	118 (88.1)	25 (73.5)	114 (83.2)	55 (71.4)	84 (89.4)
		** < 0.001**		0.195		***0.003***	
What is the main treatment choice in ≥ 75% of cases in patients presenting with 4–10 brain metastases and a GPA > 2.5?							
SRS or fSRT	53 (31.0)	21 (56.8)	32 (23.9)	13 (38.2)	40 (29.2)	32 (41.6)	21 (22.3)
WBRT ± boost or HCS	118 (69.0)	16 (43.2)	102 (76.1)	21 (61.8)	97 (70.8)	45 (58.4)	73 (77.7)
		** < 0.001**		0.308		***0.007***	
If you perform Whole-Brain-Irradiation for 4 to 10 brain metastases. do you choose a hippocampal avoidance technique?							
All cases	21 (12.6)	6 (16.2)	15 (11.5)	1 (3.1)	20 (14.8)	12 (15.6)	9 (10.0)
Not regularly, only individual cases	72 (43.1)	19 (51.4)	53 (40.8)	15 (46.9)	57 (42.2)	36 (50.6)	33 (36.7)
None	74 (44.3)	12 (32.4)	62 (47.7)	16 (50.0)	58 (43.0)	26 (33.8)	48 (53.3)
		0.251		0.199		***0.039***	
When do you schedule first clinical evaluation after radiotherapy?							
Within 1 month after RT	33 (26.0)	11 (33.3)	22 (23.4)	8 (34.8)	25 (24.0)	11 (17.5)	22 (34.4)
Within second months after RT	75 (59.1)	17 (51.5)	58 (61.7)	12 (52.2)	63 (60.6)	40 (63.5)	35 (54.7)
Within third month after RT	18 (14.2)	5 (15.2)	13 (13.8)	3 (13.0)	15 (14.4)	11 (17.5)	7 (10.9)
Within fourth month after RT	1 (0.8)	0 (0.0)	1 (1.1)	0 (0.0)	1 (1.0)	1 (1.6)	0 (0.0)
		0.629		0.727		0.118	
When do you schedule first diagnostic evaluation after radiotherapy?							
Within one month after RT	5 (3.9)	2 (6.1)	3 (3.1)	1 (4.3)	4 (3.8)	3 (4.7)	2 (3.1)
Within second months after RT	48 (37.2)	13 (36.5)	35 (36.5)	9 (39.1)	39 (36.8)	22 (34.4)	26 (40.0)
Within third month after RT	75 (58.1)	18 (54.5)	57 (59.4)	13 (53.5)	62 (58.5)	38 (59.4)	37 (56.9)
Within fourth month after RT	1 (0.8)	0 (0.0)	1 (1.0)	0 (0.0)	1 (0.9)	1 (1.6)	0 (0.0)
		0.790		0.965		0.673	
When do you switch to longer follow-up intervals after radiotherapy (with stable findings)?							
Already within 1 year	5 (4.3)	0 (0.0)	5 (6.1)	0 (0.0)	5 (5.2)	4 (6.6)	1 (1.8)
After at least 1 year	44 (37.6)	3 (8.6)	41 (50.0)	10 (47.6)	34 (35.4)	16 (26.2)	28 (50.0)
After at least 2 years	61 (52.1)	27 (77.1)	34 (41.5)	8 (38.1)	53 (55.2)	37 (60.7)	24 (42.9)
After at least 3 years	7 (6.0)	5 (14.3)	2 (2.4)	3 (14.3)	4 (4.2)	4 (6.6)	3 (5.4)
		< 0.001		0.128		0.051	
What imaging do you use when radiation necrosis is suspected? (multiple answers)							
T1 ± Gad, FLAIR	84 (71.8)	25 (69.4)	59 (72.8)	19 (79.2)	65 (69.9)	42 (77.8)	42 (66.7)
Diffusion-weighted MR	62 (53.0)	20 (55.6)	42 (51.9)	15 (62.5)	47 (50.5)	24 (44.4)	38 (60.3)
Dynamic MR sequences	24 (20.5)	10 (27.8)	14 (17.3)	7 (29.2)	17 (18.3)	10 (18.5)	14 (22.2)
MR spectroscopy	30 (25.6)	12 (33.3)	18 (22.2)	5 (20.8)	25 (26.9)	12 (22.2)	18 (28.6)
Amino-acid based PET examination	56 (47.9)	29 (80.6)	27 (33.3)		9 (37.5)		20 (37.0)
		** < 0.001**		0.421		0.068	
Hypothetical patient scenario: 55 years old, 8 BM, primary tumor NSCLC, estimated survival > 6 months, therapeutic approach?							
SRS or fSRT	55 (33.1)	21 (56.8)	34 (26.4)	11 (34.4)	44 (32.8)	33 (44.0)	22 (24.2)
WBRT approach	111 (66.9)	16 (43.2)	95 (73.6)	21 (65.6)	90 (67.2)	42 (56.0)	69 (75.8)
		***0.001***		0.868		***0.007***	
Hypothetical patient scenario: 55 years old, 8 BM, primary tumor NSCLC, estimated survival < 6 months, therapeutic approach?							
SRS or fSRT	27 (16.4)	9 (24.3)	18 (14.2)	4 (12.5)	23 (17.4)	18 (24.0)	9 (10.1)
WBRT approach	137 (83.6)	28 (75.7)	109 (85.8)	28 (87.5)	109 (82.6)	57 (76.0)	80 (89.9)
		0.143		0.500		***0.017***	
Would your decision regarding the therapy sequence change if the tumor would show EGFR/ALK mutation?							
Proceed with upfront RT	98 (71.0)	25 (73.5)	73 (70.2)	20 (71.4)	78 (70.8)	48 (75.0)	50 (67.6)
Delay RT for sole upfront TKI therapy	40 (29.0)	9 (26.5)	31 (29.8)	8 (28.6)	32 (29.1)	16 (25.0)	24 (32.4)
		0.139		0.957		0.921	
Hypothetical patient scenario: 45 yo, 15 BM, primary tumor NSCLC, estimated survival > 6 months, therapeutic approach?							
SRS or fSRT	18 (10.9)	6 (16.7)	12 (9.3)	4 (12.9)	14 (10.4)	13 (17.3)	5 (5.6)
WBRT approach	147 (89.1)	30 (83.3)	117 (90.7)	27 (87.1)	120 (89.6)	62 (82.7)	85 (94.4)
		0.210		0.693		***0.016***	
Hypothetical patient scenario: 45 yo, 15 BM, primary tumor NSCLC, estimated survival < 6 months, therapeutic approach?							
SRS or fSRT	6 (3.7)	2 (5.6)	4 (3.2)	1 (3.2)	5 (3.8)	5 (6.8)	1 (1.1)
WBRT approach	155 (96.3)	34 (94.4)	121 (96.8)	30 (96.8)	125 (96.2)	69 (93.2)	86 (98.9)
		0.511		0.870		0.061	
Would your decision regarding the therapy sequence change if the tumor would show EGFR/ALK mutation?							
Proceed with upfront RT	98 (71.5)	26 (76.5)	72 (69.9)	19 (70.4)	79 (71.8)	51 (69.9)	47 (73.4)
Delay RT for sole upfront TKI therapy	39 (28.5)	8 (23.5)	31 (30.1)	8 (29.6)	31 (28.2)	22 (30.1)	17 (26.6)
		0.462		0.881		0.644	

*RT* radiotherapy, *RPA* recursive partitioning analysis, *GPA* graded prognostic assesment, *yo* years old, *NSCLC* non-small-cell lung cancer, *EGFR* epidermal-growth-factor receptor, *ALK* anaplastic lymphoma kinase

*p-value according to Chi-Square-Test

### Prognostic scores

**Fig. 1:**
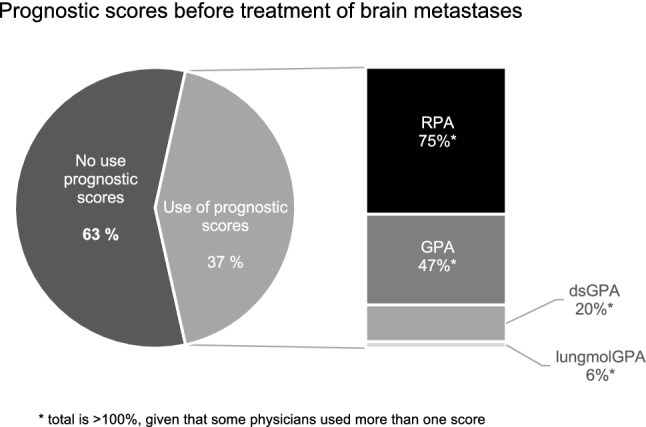
Prognostic scores

A prognostic score like Recursive Partitioning Analyses (RPA) [14], Graded Prognostic Assessment (GPA) [15], dsGPA [16] or molecularGPA [17, 18] is regularly used by 38% of the respondents before treatment of brain metastases. Those physicians who used a prognostic score selected one or a combination of these scores. The most frequently used scores were RPA in 75% and GPA in 47% (total is > 100%, given that some physicians used more than one score; Fig. 1). A significant difference in the use of prognostic scores was found between academic and non-academic centers (p-value = 0.018). Academic centers used significantly more often prognostic scores before treating brain metastases than non-academic centers. Around 37% of the physicians stated that the prognostic score influenced their treatment decision in patients with BM. Again, a statistically significant difference could be found between academic and non-academic centers (p-value = 0.014).

With respect to individual consideration of stereotactic radiotherapy in the context of prognostic scores, most radiation oncologists chose to treat patients with SRS or SRT only when presenting with “good scores”. Of those respondents who use a score the majority would select SRS only in patients with “RPA class I” or “RPA class I and II” (added together 82%) or with GPA classification in GPA > 2.5 (62%).

### Treatment approaches and management in multiple brain metastases

Radiation oncologists were also asked for their primary treatment approach, i.e. treatment decision in ≥ 75% of the cases, for patients with 4 to 10 brain metastases with GPA < 2.5. A WBRT approach was predominantly chosen (81%) either with or without boost. 19% would choose a stereotactic approach, either with radiosurgery (SRS) or a hypofractionated stereotactic radiotherapy (fSRT). When the GPA was considered as ≥ 2.5, more physicians (31%) would use a stereotactic approach (again either as SRS or fSRT) but the majority (69%) is still adhering to an WBRT approach. Results between academic vs. non-academic centers and high- vs- low-volume centers showed significant differences (see Table 2).

Only 13% of the respondents would choose a hippocampal sparing therapeutic WBRT for all patients without metastatic spread to the hippocampal region. 43% would choose the sparing technique in selected cases and 44% never use a hippocampal avoidance approach. Treatment policies regarding hippocampal sparing techniques were different between high- and low-volume centers. High-volume centers consider hippocampal sparing significantly more often than low-volume centers (p-value = 0.039). A difference between academic and non-academic centers could not be found.

When the participants were asked about the prescription doses, free text answers were relatively uniform, depending on the size of the metastases. However, when asked about the dose and isodose for an uncritical localization of metastases at size < 1 cm, no uniform answer was supplied. The most frequently given answer was 20 Gy to the 80% isodose by 21% of the participants.

### Follow-Up

Follow-up policies were relatively homogeneous and no differences between different types of centers could be found with statistical analysis. The majority (85%) schedule first clinical evaluation in the first or second month after RT, whereas 14% schedule first clinical evaluation within the third month. The majority (> 95%) schedule first imaging within the second or third month after radiotherapy. A small part (4%) would even go for first imaging within the first 4 weeks after radiotherapy. The only difference could be found between academic and non-academic centers in the selection of the time to switch to a longer period between the individual follow-up times. The majority of academic centers (77%) would only switch to a longer follow-up interval after at least 2 years and 14% even after 3 years with a stable course. Whereas the majority of non-academic centers (56%) would switch to a longer interval between the follow-up examinations already after 1 year.

If radiation necrosis was suspected most radiation oncologists would rely on results of T1 ± Gadolinium and FLAIR examinations (71%). Amino-acid based PET examinations were chosen by 81% of respondents out of academic centers, whereas non-academic centers consider PET examinations only in 33%. The policy regarding diagnostic examinations in suspected radiation necrosis was statistically different between academic and non-academic centers (p-value < 0.001).

### Scenarios of patients presenting with multiple BM

The last questions of the survey consisted of specific clinical scenarios of patients with BM where a sample of BM spread was illustrated. The first case was a 55-year-old patient with 8 newly diagnosed BM of NSCLC origin. All BM were < 5 mm^3^ in size and in non-eloquent locations regarding organs at risk, such as optic chiasm or brainstem. In case of a median survival more than 6 months, 32% would choose a stereotactic approach, either as a SRS or fSRT, 68% would select an WBRT approach. Treatment policies differed between academic and non-academic centers (p-value ≤ 0.001) and also between high- and low-volume centers (p-value = 0.007). When the median survival is estimated to be less than 6 months 15% would still choose a stereotactic approach, while the majority would choose a WBRT approach (See Table 2). A difference in the treatment policy could be detected again between high- vs. low-volume centers with high volume centers preferring less WBRT.

The second case was a 45-year-old patient with 15 newly diagnosed BM of NSCLC, all were < 5 mm^3^ in size and in non-eloquent locations. In case of a median survival more than 6 months, 16% of the respondents would choose a stereotactic radiotherapy approach. Statistically different treatment policies were apparent between academic and non-academic centers (p-value = 0,009) and also between high- and low-volume centers (p-value = 0,001), with both—academic and high-volume centers—being more open to a stereotactic radiotherapy approach. With an estimated survival of less than 6 months more than 95% of the respondents would choose a WBRT approach and no statistical difference could be detected.

**Fig. 2:**
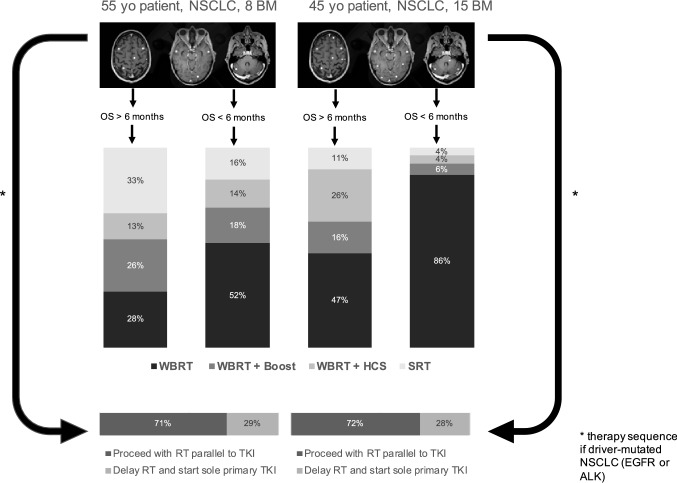
Radiotherapy approaches in hypothetical patient cases with multiple brain metastases of NSCLC and decision on therapy sequence when targeted therapy is available in driver-mutated NSCLC

After participants were asked about primary treatment for visualized distribution of brain metastases from NSCLC for specific estimated survival times in both cases, they were additionally asked whether the decision to use appropriate upfront radiotherapy would change if a driver mutation with TKI systemic therapy were present. More than two thirds (71% first case, 72% second case) would not omit up-front radiotherapy for sole TKI therapy, and around one third would choose sole initial TKI therapy and delay radiotherapy. No difference in institution-dependent or position-dependent treatment strategies could be found. All chosen treatment approaches for each case and the decision regarding therapy sequence in driver-mutated NSCLC are summarized in Fig. 2.

## Discussion

With almost 200 respondents, our survey presents the first collected data on general management and patterns of care in patients with multiple BM in Germany. Other surveys on the management of BM were not specific to multiple BM and mostly distributed among centers in English speaking countries and Japan [19–22]. To our knowledge, only one European survey focusing on the management of brain metastases of non-small-cell lung cancer exists [23].

Interestingly, the vast majority of radiation oncologists would select conventional WBRT as the first choice for patients with multiple BM, even in patients with good prognostic factors. Only a lower proportion considered SRS in patients with more than 4 BM, especially when presenting with good prognostic factors. The results of our survey reflect the ambiguity of national and international guidelines in the selection of patients suitable for stereotactic radiotherapy. The guidelines give a broad scope for the choice of an appropriate therapy for multiple brain metastases. Regarding stereotactic radiotherapy, the majority of participants seem to adhere to clear instructions of the guidelines and do not regularly treat stereotactically beyond the cut-off of 4 metastases. Noteworthy, our survey additionally revealed that hippocampal sparing WBRT is only used by a few participants on a regular and consistent basis.

Early research findings indicating SRS and hippocampal sparing techniques in multiple brain metastases have already been available for a long time before our survey was initiated [24–27]. In contrast, our survey has shown that WBRT without hippocampal avoidance still dominates chosen treatment approaches in the setting of multiple brain metastases. Recently published results of phase 3 trials strengthen the use of stereotactic radiotherapy or the use of hippocampal sparing techniques [28, 29]. A randomized controlled phase 3 trial conducted by the MD Anderson Cancer Center has shown equivalent survival and significantly less cognitive decline when comparing SRS to WBRT in 4 to 15 brain metastases [28]. For hippocampal sparing the NRG-CC001 trial delivered the highest evidence to date with randomized comparison of WBRT plus memantine with and without hippocampal avoidance [29]. A better preservation of patients' cognitive function in patients receiving WBRT with hippocampal avoidance and memantine could be shown. Nevertheless, questions regarding the optimal treatment modality in the setting of multiple brain metastases have not been conclusively clarified and the appropriate treatment for multiple brain metastases still remains ambiguous. For hippocampal-avoidant WBRT long-term effects are still unclear [30, 31]. For SRS in multiple brain metastases the question for eligible histologies is unanswered as the MD Anderson study excluded melanoma patients, which can often present with multiple brain metastases [28]. Therefore comprehensive high clinical evidence for the treatment of multiple brain metastases is still lacking. The results of further ongoing randomized trials comparing (HA-) WBRT and SRS for patients with multiple BM are expected and will hopefully better define the role of different treatment approaches in this setting [32]. A phase III trial (NCT03550391) will directly compare SRS against hippocampal-avoidant WBRT plus memantine in patients with 5 to 15 brain metastases and study completion is awaited for mid of 2022. In general, conventional WBRT is more and more being challenged by slowly changing pattern practice and may become obsolete with the emergence of prospective evidence.

Our statistical analysis revealed significant differences in treatment policies for multiple brain metastases between academic and non-academic centers or between high- and low-volume centers. Academic centers and high-volume centers seem to have already adapted to the recent increasing evidence of stereotactic radiotherapy in the setting of multiple metastases or choose a hippocampal sparing technique with whole brain irradiation more often.

In general, our survey demonstrates a restrictive use of prognostic scores before treatment of brain metastases. The results of our survey provide a pattern that prognostic scores are applied more frequently in academic centers than in non-academic centers. Additionally, treatment recommendations for cerebral irradiation also seem to be based more often on interdisciplinary tumor board decisions in academic centers, which is also evident for high-volume centers compared to low-volume centers. With an increase in therapeutic options for patients with oncological diseases, further development of existing treatments and the introduction of new systemic substances, especially monoclonal antibodies and other targeted substances, the oncological therapy landscape has diversified enormously in recent years. Due to the complexity of oncological diseases and numerous possibilities, interdisciplinary tumor boards have become indispensable and should be demanded as a standard in oncological care by all professional societies with the aim of finding the most promising individual therapy.

The question regarding the optimal integration of promising cerebral systemic therapy was addressed in our specific patient cases. Nearly one-third of respondents would initially favor systemic therapy with TKI alone in newly diagnosed driver-mutated NSCLC with multiple brain metastases and defer cerebral radiotherapy. The question of integration of systemic therapy in driver-mutated NSCLC was addressed in several retrospective studies [33, 34]. The deferral of radiotherapy was associated with inferior outcome in terms of OS and different meta-analyses of retrospective studies strengthened the hypothesis of improved OS by up-front SRS [35–37], although prospective evidence on this open question is still missing. Up to now, there is no randomized trial comparing sole upfront TKI-therapy with upfront cranial radiotherapy followed by TKI-therapy. It should be noted that newer generation TKIs demonstrate superior intracranial efficacy [38, 39].

The patient cases illustrate another peculiarity. While some participants consistently use WBRT for multiple metastases, in others the classic cut-off for stereotactic radiotherapy seems to be far off, and stereotactic radiotherapy is also used for far more than 10 metastases.

We realize that our survey has some limitations: with the rapid emergence of technological improvements in radiation oncology and the evolution of systemic therapies with increasing brain activity for different tumor types, a tumor-agnostic approach to BM has to be viewed with caution. We tried to focus on the relevant topics and to allow the response to the full survey in around 10 to 15 min, as it is known that the number of respondents decreases when the number of questions and time to survey completion increases [40]. Since we specified in the survey only the number of metastases and not the volume, we received no information about the total intracranial tumor volume which may be considered for a SRS approach. In addition we could not further differentiate between different histologies except in the fictitious patient cases on NSCLC. Furthermore, the whole spectrum of immunotherapy options for brain metastases and integration of information about tumor mutational burden was not discussed in our questionnaire and represents another limitation as well as further possible treatment approaches for brain metastases e.g. laser interstitial thermal therapy (LITT). A possible response bias should not be underestimated and represents another limitation.

Despite these caveats, as our survey was answered mostly by experienced radiation oncologists from a broad variety of institutions including private practices, we consider the responses representative and quite homogenous in their distributions and that the survey covers a representative picture within German speaking countries.

## Conclusion

Our survey provides a comprehensive overview of the patterns-of-care of multiple BM among German radiation oncologists. Patient selection is still not fully based on prognostic scores, as they were only used in selected cases to guide treatment decisions. Among different types of centers we see great differences in treatment policies. Interestingly, WBRT without hippocampal avoidance remains the treatment of choice in the vast majority of patients with multiple BM, despite emerging evidence of data for the safety and effectiveness of stereotactic radiotherapy treatment or better preservation of neurocognition with dose reduction to the hippocampal stem cell compartment. Regarding the integration of system therapies in driver-mutated lung cancer, different strategies exist with a considerable proportion preferring upfront systemic therapy with TKI alone. Prospective trials are needed to assess the optimal timing of radiation for patients with BM who are candidates for CNS-active systemic therapies, to determine whether SRS can be safely delayed without affecting survival and neurocognition.

While management of multiple BM becomes increasingly important, the need for updated guidelines and recommendations is evident.
